# The Regulatory Role of Mitochondrial MicroRNAs (MitomiRs) in Breast Cancer: Translational Implications Present and Future

**DOI:** 10.3390/cancers12092443

**Published:** 2020-08-28

**Authors:** Miguel A. Ortega, Oscar Fraile-Martínez, Luis G. Guijarro, Carlos Casanova, Santiago Coca, Melchor Álvarez-Mon, Julia Buján, Natalio García-Honduvilla, Ángel Asúnsolo

**Affiliations:** 1 Department of Medicine and Medical Specialities, Unit of Histology and Pathology, Faculty of Medicine and Health Sciences, University of Alcalá, 28801 Alcalá de Henares, Spain; oscar.fraile@edu.uah.es (O.F.-M.); carlos.casanovam@edu.uah.es (C.C.); s.coca@uah.es (S.C.); melchor.alvarezdemon@uah.es (M.Á.-M.); mjulia.bujan@uah.es (J.B.); natalio.garcia@uah.es (N.G.-H.); 2 Ramón y Cajal Institute of Sanitary Research (IRYCIS), 28034 Madrid, Spain; angel.asunsolo@uah.es; 3 Cancer Registry and Pathology Department, Hospital Universitario Principe de Asturias, 28806 Alcalá de Henares, Madrid, Spain; 4 Department of System Biology, Unit of Biochemistry and Molecular Biology (CIBEREHD), University of Alcalá, 28801 Alcalá de Henares, Spain; luis.gonzalez@uah.es; 5 Immune System Diseases-Rheumatology, Oncology Service an Internal Medicine, University Hospital Príncipe de Asturias, (CIBEREHD), 28806 Alcalá de Henares, Madrid, Spain; 6 Department of Surgery, Medical and Social Sciences, Faculty of Medicine and Health Sciences, University of Alcalá, 28801 Alcala de Henares, Madrid, Spain

**Keywords:** breast cancer, microRNAs, mitochondria, mitomiR, translational medicine

## Abstract

**Simple Summary:**

Mitochondrial microRNAs (mitomiRs) are an emerging field of study in a wide range of tumours including breast cancer. By targeting mitochondrial, or non-mitochondrial products, mitomiRs are able to regulate the functions of this organelle, thus controlling multiple carcinogenic processes. The knowledge of this system may provide a novel approach for targeted therapies, as potential biomarkers or helping in the diagnosis of such a complex malignancy.

**Abstract:**

Breast cancer is the most prevalent and incident female neoplasm worldwide. Although survival rates have considerably improved, it is still the leading cause of cancer-related mortality in women. MicroRNAs are small non-coding RNA molecules that regulate the posttranscriptional expression of a wide variety of genes. Although it is usually located in the cytoplasm, several studies have detected a regulatory role of microRNAs in other cell compartments such as the nucleus or mitochondrion, known as “mitomiRs”. MitomiRs are essential modulators of mitochondrion tasks and their abnormal expression has been linked to the aetiology of several human diseases related to mitochondrial dysfunction, including breast cancer. This review aims to examine basic knowledge of the role of mitomiRs in breast cancer and discusses their prospects as biomarkers or therapeutic targets.

## 1. Current Perspectives of Breast Cancer

Breast cancer is the most frequently diagnosed female neoplasm in the world, where one woman in every eight will develop this disease [1]. Despite being the leading cause of cancer-related mortality, 5-year survival rates in high and middle-income countries are on the rise because of advances in the molecular understanding and clinical management of breast cancer, as well as in screening and early diagnosis methods. However, these statistics seem to be reversed in less developed countries where the chances of having access to these resources are much lower [2,3]. Breast cancer can also affect men, albeit with an incidence of less than 1% of all cases of breast cancer and featuring different biological characteristics than in women [4]. For reasons as yet unknown, the incidence of this disease in men is increasing, therefore tumours in this population also need be considered in the management of breast cancer.

Among the most important risk factors are an older age, family history, reproductive factors such as early menarche, late menopause or nulliparity, hormone factors (oestrogen levels), and life-style related factors such as physical inactivity, obesity, alcohol consumption and exposure to tobacco smoke [5,6,7,8].

Breast cancer may be studied by their molecular profile, based on the presence or absence of three receptors: the oestrogen receptor (ER), progesterone receptor (PR) and epidermal growth factor receptor Her2/Neu. Accordingly, the luminal subtypes A and B are both ER/PR+. Luminal A tumours are characterised by being Her2- and featuring low levels of the proliferation marker Ki67, while luminal B shows high levels of Ki67 and is Her2+. There is also a subtype Her2+ ER/PR- and triple negative tumours (TNBC), which lack all three receptors [9,10]. Each subtype also appears in different frequency and is linked to a different survival rate. Luminal A accounts for 30–40% of all invasive tumours and is associated with a better prognosis. Luminal B tumours appear at a frequency of 20–30% and show a worse prognosis; Her2+ (12–20%) are aggressive tumours yet respond well to treatments directed at inhibiting this receptor, such as herceptin (trastuzumab); and TNBC, which make up the remaining 15–20%, show the worse prognosis and are the most difficult to treat [11]. Interestingly, some authors distinguish in TNBC tumours a predominant basal-like phenotype (70–80%), which is more malignant, and a less frequent non-basal like type, this criterion being especially useful to predict survival in these patients [12]. In addition, approximately 95% of breast tumours diagnosed in men are ER+/PR+, suggesting a possible role of the oestrogen receptor in the biology of male breast tumours [13].

Currently, patients with breast cancer are benefiting very positively from the introduction of more specific molecular therapeutic targets. Among some examples, we should highlight the impact of endocrine therapy for ER+ tumours using aromatase inhibitors or tamoxifen, or the antibody herceptin/trastuzumab for tumours showing HER2+ overexpression [14]. PI3K/Akt inhibitors have also reported a great potential in the clinical management of the different subtypes of tumours [15]. The more aggressive tumours like TNBC require other targets for their treatment such as inhibitors of immune checkpoints, platinum compounds, PARP or PI3K inhibitors, and even androgen receptor inhibitors [16].

In this context, microRNAs are emerging as a potential biomarker and therapeutic target for a wide variety of tumours in which their dysregulation forms part of their carcinogenic mechanism [17,18,19], hence representing a promising point of study in breast cancer.

## 2. MicroRNAs in Breast Cancer

MicroRNAs are a class of small non-coding RNAs of approximately 22 nucleotides that are synthesized and processed in the cell nucleus and cytoplasm. MicroRNA biogenesis starts with the actions of RNA polymerase II/III to form a primary microRNA (pri-microRNA). This primary transcript is then processed and cut by a ribonuclease III enzyme Drosha and an RNA-binding protein DiGeorge Syndrome Critical Region 8 (DGCR8) to give rise to a microRNA precursor (pre- microRNA), which is exported to the cytoplasm by exportin 5 (XPO5) [20,21]. Once in the cytoplasm, the RNase III endonuclease Dicer converts pre-microRNA into a double-stranded microRNA (ds-microRNA), which forms part of an RNA-induced silencing complex (RISC), composed of Dicer, trans-activation response RNA-binding protein (TRBP) and a member of the Argonaute (AGO) family of proteins (AGO1-4 in humans) [22]. TRBP contains three RNA-binding domains which recognize the guide and passenger strands of ds-microRNA and forms a duplex with Dicer. Knock-out models have shown that the formation of this Dicer-TRBP complex is essential for microRNA biogenesis [23]. Once these strands have been recognized, AGO is able to eliminate the passenger strand while maintaining the guide strand that will make up the mature microRNA known as miRISC.

MicroRNAs control a wide variety of biological processes through their posttranscriptional regulatory role in gene expression [24]. This effect is produced via their binding to the microRNA response element (MRE), which is mainly located in the 3′UTR region of messenger RNA (mRNA), although this response element can also be found in the 5′UTR region or even in codifying regions [25,26]. The binding of miRISC to mRNA suppresses gene expression by two main mechanisms: hybridizing with mRNA and degrading it or inhibiting the translation of mRNA [27]. Studies have shown that some microRNAs can bind to an already expressed protein, directly modifying its function [28]. A single microRNA can regulate around 200 different transcripts and a single mRNA can be controlled by several different microRNAs [29]. MicroRNAs represent around 2–3% of the human genome, approximately half of which are grouped into clusters, with transcripts like polycistrons generally having two mature microRNAs per cluster [30]. Regulation via microRNA is a very evolutionary conserved process and over 60% of genes coding for human proteins are susceptible to their control [31].

In cancer, microRNAs can act both as tumour suppressors (tsmicroRNAs) inhibiting the expression of oncogenes or contrarily may inhibit tumour suppressor genes in which case they are known as oncomicroRNAs. Several mechanisms have been described whereby the tumour cell can modulate microRNA concentrations, for example at the level of their biogenesis or exporting to the cytoplasm [32,33]. Further insight is needed into these mechanisms whereby tumour cells control the expression of microRNAs to identify new therapeutic targets specific to these alterations.

MicroRNAs constitute a major epigenetic mechanism and their implications in breast cancer have extensively been studied [34,35]. For instance, it is known that microRNAs play a critical role in breast cancer by their interplay with BRCA1 and BRCA2 [36]. Also, microRNAs dysregulation has been associated with cell proliferation, apoptotic response, metastasis, or cancer recurrence in breast cancer, amongst others [37]. The cytoplasm is the most common localization of microRNAs in the cell, where cytosolic mRNAs are regulated. However, the important role of microRNAs in other cell compartments has also been described. For instance, it is known that microRNAs may be found in the nucleus, whereby they bind to the promotor or potentiating regions of some genes, thus controlling their epigenetic cell regulation [38]. Here, it is known that importin 8 (IPO8) is essential for transporting mature microRNAs to the nucleus [39]. MicroRNAs have also been identified in the endoplasmic reticulum, where they seem to play a key role in controlling endoplasmic reticulum stress [40]. Finally, the mitochondrion is another organelle in which microRNAs may be found, where they are known as mitomiRs [41]. The relevance of mitochondrion in cancer was firstly described by the biochemist Otto Warburg who stated that the origin of tumour cells was the damage at the mitochondrial level which was largely held responsible for their malignant transformation [42]. However, nowadays it is known that rather than being damaged, the mitochondrion is altered and that these changes play a key role in carcinogenesis in a great variety of tumours. Hanahan and Weinberg [43] described several hallmarks of cancer including the most distinctive characteristics of cancer cells such as metabolic reprogramming, apoptosis evasion, inflammation, genomic instability, or tumour metastasis. Giampazolias and Tait [44] reported the contributions of mitochondria to these hallmarks highlighting the impact of this organelle in tumour cell biology. In this background, mitomiRs arise as a vital regulator of mitochondrial functionality, and their alterations have been found in many pathologies, including breast cancer [45,46,47,48]. This review considers current understanding of the role of these mitomiRs in breast cancer and discusses their prospects as targets of adjuvant therapy or as precision biomarkers in these tumours.

## 3. Mitochondrial MicroRNAs in Breast Cancer

### 3.1. Regulatory Mechanisms of MitomiRs

MitomiRs are a group of microRNAs that appears to tightly orchestrate mitochondrion functions. Bandiera et al. [49] described a total of 57 microRNAs differentially expressed in mitochondria and cytosolic fractions. From the whole sample of microRNAs, 13 of them were found to be enriched in mitochondria purified HeLa cells, named as mitomiRs. Since then, a wide variety of mitomiRs have been described, depending on the tissue or model studied [50]. The vast majority of mitomiRs are transcribed in the nucleus, although some like miR-1974, miR-1977 and miR-1978 are coded for by mtDNA [51].

**Figure 1:**
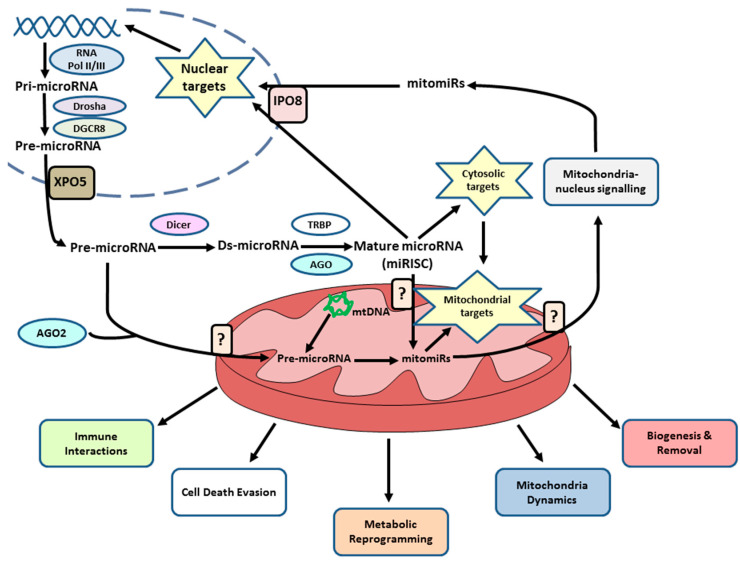
Role of microRNAs in the mitochondrion in breast cancer. As shown in the diagram, most mitomiRs are transcribed in the nucleus, although some may be coded for by mitochondrial DNA. The processing of microRNA takes place in the nucleus and cytoplasm through the actions of several proteins, although different studies indicate that some mitomiRs could finish their processing in the mitochondrion. Mature or precursor microRNA may be imported into the mitochondrion although the precise mechanisms for this are still unknown. Once processed, mitomiRs directly mediate a series of mitochondrial products, regulating post transcriptionally any mRNAs or proteins present in the mitochondrion, impairing its function. These products may be encoded by either the nuclear or mitochondrial genome. Sometimes, microRNAs indirectly modulate mitochondrial functions through their actions on products found in other cell compartments, promoting their action in the mitochondrion. Further, a single microRNA can be directed at different products outside and inside the mitochondrion. These different mechanisms give rise to a series of cancer hallmarks such as evading cell death, immune system interaction, and metabolic reprogramming as well as the regulation of a series of carcinogenic processes via their actions on the biogenesis-degradation of mitochondria, changes in mitochondrial dynamics or nucleus-mitochondrion signaling, where it is known that mature microRNA exert their actions, among them some mitomiRs involved in the epigenetic regulation of the tumour cell. Nucleus-mitochondrion communication is also important in regulating all these mitochondrial functions, even the import of microRNA to the mitochondrion.

The mechanisms whereby mitomiRs modulate mitochondrial function in cancer are highly complex (Figure 1). Evidence so far indicates they are able to regulate products located inside the mitochondrion. As the expression and regulation of mitochondrial genes takes place in the mitochondrial matrix, to exert their actions, microRNAs need to cross the external membrane, intermembrane space and internal membrane [41].

Despite mounting interest in this area of study, the mechanisms underpinning the import of mature microRNAs into the mitochondrial matrix to regulate the expression of mitochondrial genes have not been fully elucidated. Moreover, the presence of pre-microRNA and the protein AGO2 has been detected in the mitochondrion suggesting that part of the biogenesis of some mitomiRs could take place inside the mitochondrion, and that AGO2 could also intervene in their transport towards the interior [52,53,54]. In addition, the preferential localization of AGO-2 has been shown to be the mitochondrial membrane [49], which may be relevant for further mitomiRs isolation procedures.

It has also been described that, while not imported into the mitochondrion, microRNAs are able to regulate a series of cytosolic or nuclear key products for mitochondrial function, and these need to be taken into account to obtain an overview of the role played by microRNAs in the mitochondrion in breast cancer (Figure 1). In addition, some mitomiRs are shown to target multiple mitochondria mRNA, rRNA and tRNA and simultaneously nucleus-encoded mitochondria-associated mRNAs [55], thus denoting the intricacy of mitomiRs in mitochondrion regulation.

Among the functions regulated by mitomiRs in breast cancer are cell metabolism abnormalities, apoptosis evasion, mitochondrial biogenesis and degradation, mitochondrion-nucleus retrograde and anterograde signaling, mitochondrial dynamics and immune system interactions.

### 3.2. Purification, Isolation, and Detection of MitomiRs

MicroRNAs may be studied directly from cells, tissues or a wide range of body fluids including plasma, saliva, urine, tears, cerebrospinal fluid, semen, or breast milk [56]. Cells and tissues are the preferred samples to study mitomiRs, being necessary to separate the mitochondrion from other cellular compartments.

Differential centrifugation is a simple way to successfully isolate mitomiRs [57]. After this procedure, RNAse A treatment is used to degrade cytosolic microRNAs contamination and subsequently Percoll gradient may be a resource for microRNA purification and extraction. The presence of small RNA is confirmed by capillary electrophoresis, and its detection is achieved by RT-qPCR, where some algorithms like Normfinder could be applied to find mitomiRs [58].

Different strategies can be also used to detect microRNA expression, like in-situ hybridization, microarrays, and RNA-sequencing [59]. Equally important, further techniques allow the isolation of mitomiRs such as the magnetic antibody cell sorting method (MACS). It is known that the use of MACS reported the highest yield to isolate mitochondria when compared to differential centrifugation or ultracentrifugation [60].

**Figure 2:**
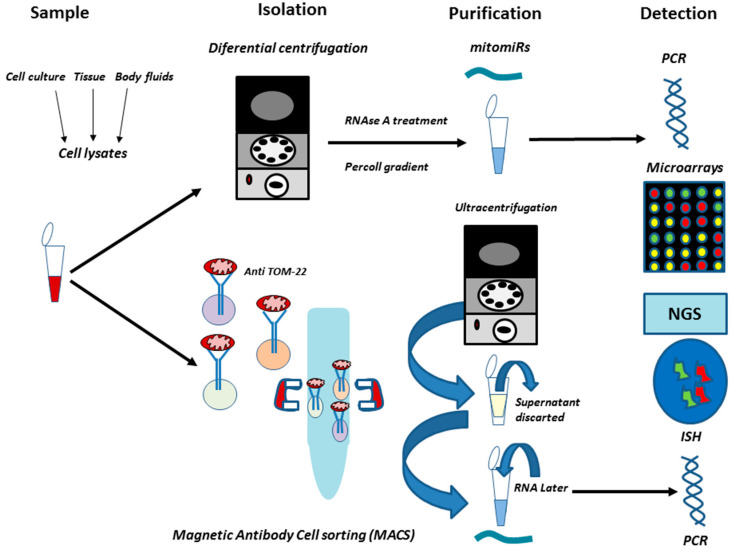
Methodological approaches to study of mitomiRs in breast cancer. Many samples may be used to isolate mitomiRs. However, cell cultures and tissues are the most indicated as cell lysates are the most feasible manner to work with mitomiRs. Different procedures have been described to isolate and purification of mitomiRs. Notwithstanding, Magnetic Antibody Cell sorting (MACS) appears to be the most appropriated technique to study mitomiRs, as functional mitochondrion may be isolated by targeting a specific protein as TOM-22. Then ultracentrifugation could be used to finally purify the sample, subsequently detected by PCR. Other methods have also been used to achieve this goal, such as microarrays, RNA sequencing or in situ Hybridization.

Barrey et al. [52] described a method to isolate mitomiR by using MACS. In summary, cells were lysed, and mitochondria were magnetically labeled with anti-TOM22 antibody microbeads. This monoclonal antibody specifically binds to the translocase of the outer mitochondrial membrane 22 (TOM22) of human mitochondria. The labeled cell lysate was loaded in a column placed in a magnetic field separator. The magnetically labeled mitochondria were retained in the column during washing. Then the magnet is removed from the column support and mitochondria were eluted. Notwithstanding the success of the method, to optimize feasible protocols to obtain reproducible results while studying mitomiRs remains necessary. Finally. multiple kits have been designed to ease the study of mitomiRs by isolating mitochondria or detecting microRNAs. However, it is important to understand that not all kits report the same results, depending on the sample, to carefully select the most suitable kit must be also beared in mind [61,62]. Likewise, considering the type of sample is also crucial, as fluids present a lower concentration of microRNA in comparison to cells or tissues. Furthermore, mitomiRs may be found extracellularly in exosomes, complexes containing Argonaute protein or even at lipoproteins [63]. Additional works will be recommended to study mitomiRs in body fluids, as cell lysates are the best manner to isolate mitomiRs (Figure 2).

### 3.3. Role of Mitochondrial MicroRNAs in Breast Cancer

#### 3.3.1. Mitochondrial MicroRNAs in Tumour Metabolism

Warburg [64] showed that tumour cells undergo transition from an aerobic energy metabolism towards a fermentation state. This metabolic reprogramming promotes the process of glycolysis and lactate formation and is known as the Warburg effect. This status continues even in the presence of oxygen such that it is also termed aerobic glycolysis [65]. By modifying its metabolism, the tumour cell induces its own proliferation through its increased capacity to synthesize new components despite the lowered energy production, which is also facilitated by increased glucose uptake [66]. The tumour cell continues to undertake cell respiration, but at a much lower rate than healthy cells. These characteristics have been targets of techniques that can detect these metabolic changes such as positron emission tomography (PET) imaging.

The regulation of cell tumour metabolism by mitomiRs occurs by various pathways. To date, evidence exists that this regulation is, in turn, controlled by different receptors, growth factors and oncogenic proteins, among which we find the PI3K/Akt pathway and the hypoxia-induced factor (HIF-1α), whose important role in glucose uptake and the process of glycolysis in tumour cells has been confirmed [67]. Tumour cells make use of some microRNA to regulate these pathways in breast cancer. An example is miR-210, whose levels increase through the actions of HIF-1α and has as its target a great variety of enzymes involved in mitochondrial metabolism, favouring the metabolic reprogramming of these tumours, evading apoptosis or participating in angiogenesis among other functions [68].

Similarly, several studies have shown that high Lin28 levels and reduced levels of the let 7 microRNA may provoke aerobic glycolysis following their actions on certain mitochondrial products such as PDK1 [69]. In effect, this mechanism has been described in TNBC tumours, whereby the combined use of doxorubicin and let 7 microRNA was observed to increase treatment sensitivity in these cell lines in vitro [70]. Recently, Li et al. [71] established the role of cancer-associated fibroblasts (CAF) in this metabolic reprogramming through the release of exosomes, in whose interior a microRNA is found, the miR-330-5p. The latter is key for the inhibition of mitochondrial oxidative phosphorylation and for promoting tumor cell growth and positively regulating the enzyme pyruvate kinase M2 (PKM2), which drives the transformation of pyruvate into lactate. Interestingly, this process is modulated by SNHG3, a long non-coding RNA (lncRNA), which favours carcinogenesis in a wide range of tumours including breast carcinoma [72].

Yuan et al. [73] reported that reduced miR-133a levels are directly involved in increasing levels of UCP-2. This molecule belongs to the family of UCPs, a set of mitochondrial anions whose overexpression has been correlated with several cancers, and also plays a major role in the metabolic reprogramming of tumor cells [74]. Similarly, it has been observed that this increase in miR-133a/UCP-2 is also associated with doxorubicin resistance in MCF-7 breast cancer cell lines [73].

In several in vitro tumor models, Jung et al. [75] identified a relationship between the nuclear factor (erythroid-derived 2)-like 2 (NFE2L2; NFE2L2/NRF2), a master regulator of the antioxidant response that is highly implicated in multiple hallmarks of cancer [76], and the mitochondrion. These authors observed how its silencing promotes the overexpression of miR-181c, thus inhibiting the expression of MTC01, subunit 1 of cytochrome C oxidase, complex IV of the ETC. In addition, their results also showed how miR-181c/MTCO1 is also related to the activation of AMPK, a molecule involved in metabolic reprogramming and cell signaling among other processes in breast cancer [77]. Indeed, the inhibition of this microRNA is a promising therapeutic target in these tumours.

Recently, Peng et al. [78] reported the role of miR-3677 overexpression in breast cancer cells through its blocking effect on transducin-like enhancer of split3 (TLE3). Pearson et al. [79] noted that TLE3 loss correlated with enhanced glucose uptake in adipose tissue and also induced a series of metabolic changes in the mitochondrion. Accordingly, tumour cells could use this mechanism for their metabolic reprogramming.

In addition, modifications have been also described in other metabolic pathways in which mitomiRs play a key role, such as in the case of melatonin. Melatonin has proved to be a potential therapeutic agent against several cancers including breast [80,81]. The important role of melatonin has been confirmed in several cohort studies, in which lower melatonin levels caused by alterations in the circadian rhythm was identified as a risk factor for breast cancer [82,83]. Mitochondria have the capacity to synthesize and release melatonin to the cytoplasm. Moreover, this release seems to have an “automitocrine” effect, that in other words, it has the ability to signal to the mitochondrion itself via heterodimerization of G protein-coupled receptors (GPCR) [84]. Melatonin has a toxic effect on breast cancer cells and descriptions exist of its modulating role in the respiratory chain, increasing the release of mitochondrial calcium, and in promoting apoptosis [85]. Recently, Anderson [86] described that tumour cells rely on, among other components, microRNAs such as miR-7, miR-375 or miR-451, whose downregulation increases the formation of N-acetylserotonin (NAS), the precursor of melatonin. An increased NAS/melatonin ratio greatly benefits tumour cells, as melatonin levels are reduced and kinase tyrosine receptor B is activated by NAS thus promoting their survival. More work is needed to address the different mechanisms used by tumour cells as targets for more effective therapeutic strategies against breast cancer.

Finally, the role of mitomiR in other metabolic pathways has also been described. Such is the case of miR-195, a tumour suppressor microRNA that appears diminished in invasive breast cancer [87]. Singh et al. [88] discovered the role of miR-195 in the inhibition of the protein cytochrome P450 family 27, subfamily B, polypeptide 1 (CYP27B1), occurring in the internal mitochondrial membrane, where it is involved in vitamin D metabolism. CYP27B1 has been found overexpressed in a differential way in the tissues of patients with breast cancer compared with control subjects [89] suggesting a role of this cytochrome in carcinogenesis. Other studies have shown that miR-195 also regulates a wide variety of enzymes that control lipogenic pathways, such as acetyl-CoA carboxylase 1/alpha (ACACA) and fatty acid synthase (FASN), key enzymes in the synthesis of fatty acids, or 3-hydroxy-3-methylglutaryl-CoA reductase (HMGCR), which drives cholesterol metabolism. These products have also been assessed as therapeutic targets for the treatment of some breast tumours [90,91,92].

#### 3.3.2. Mitochondria, MicroRNAs, and Cell Death Evasion

Mitochondria play a key role in the capacity tumour cells have of avoiding death and are especially implicated in evading apoptosis [93]. One of the most significant events in the process of apoptosis mediated by mitochondria is the formation of a mitochondrial permeability transition pore (mPTP). This structure leads to loss of mitochondrial membrane potential and the exit of some of its components through a process known as mitochondrial outer membrane permeabilization (MOMP) [94]. It has been proposed that released mitochondrial products such as cytochrome C bind to different signaling proteins, in this case, the apoptotic protease activation factor 1 (APAF-1), which in turn activates caspases, a key set of proteases for apoptosis [95]. Interestingly, Ichim et al. [96] described a special process, they termed “minority MOMP” whereby activation of caspases was insufficient to initiate the process of apoptosis but was responsible for DNA damage inducing genome destabilization, and with it, malignant transformation and tumorigenesis.

The control of MOMP in mitochondria is mediated by proteins of the family Bcl-2, which may be proapoptotic or antiapoptotic. The different proteins of this family share a Bcl-2 domain that is highly conserved. BH3-Only and BAX/BAK are notable subfamily members comprised in the proapoptotic proteins while Bcl-2 is often used to refer the antiapoptotic members [97]. After a proapoptotic stimulus, BH3 proteins are only able to promote the activation of BAX and BAK, which launch the process of mitochondrial membrane permeabilization and then, the entry on apoptosis. Activation of Bcl-2 antiapoptotic protein family lead these antiapoptotic members the binding to BH3-Only or to BAX and BAK, thus blocking apoptosis [98].

Tumour cells use different mechanisms to evade apoptosis, for example, by modulating the actions of these pro/antiapoptotic proteins. Zhou et al. [99] examined by RT-qPCR, the expression of miR-27a in cell lines and tissue specimens from patients with breast cancer. Their results revealed inverse correlation between levels of miR-27a and the protein BAK, with the capacity to modulate cell proliferation and metastasis. Interestingly, the authors also described how mice knockouts for miR-27a showed considerably increased sensitivity to cisplatin treatment, promoting apoptosis by the mitochondrial pathway. They therefore demonstrated the important role of this microRNA in driving mitochondrial apoptosis and in resistance to certain treatments. In this same line, Sharma and Kumar [100] revealed the existence of some oncomicroRNAs like miR-21 or miR-155 involved in evading this process. Further, they showed how treatment with metformin reduced and modulated levels of these microRNAs and of antiapoptotic proteins such as Bcl-2, or proapoptotic ones such as BAX, thus inducing apoptosis in vitro.

HAX-1 belongs to the subfamily Bcl-2 of antiapoptotic proteins found located in the mitochondria, and their overexpression has been reported in breast cancer cells resistant to cisplatin and doxorubicin [101]. In these cells, levels of miR-100 are reduced and when their overexpression is induced, the susceptibility of these cells to treatment with cisplatin increases significantly because of the inhibitory effect on HAX-1 [102]. Sun et al. [103] showed that by inducing the overexpression of miR-223 in TNBC cell lines, sensitivity to apoptosis induced by tumour necrosis factor-related apoptosis-inducing ligand (TRAIL) was also promoted through its action on HAX-1. Effectively, this is an important factor to consider in the therapy of these tumours.

Several in vitro studies have shown that the downregulation of miR-125b favours the evasion of apoptosis by breast cancer cells and is also involved in the resistance these cells have to doxorubicin [104,105]. Their results reveal that combined treatment miR-125b/ doxorubicin leads to loss of the mitochondrial membrane’s potential and to the process of MOMP, thus inducing the activation of caspase. In parallel, Xie et al. [106] detected via real time PCR a significant reduction in miR-519d in breast cancer stem cells resistant to cisplatin. The combined treatment of cisplatin and this microRNA increased the sensitivity of the cells to treatment. This effect was achieved through its binding to MCL-1, an antiapoptotic protein. Further, Singh et al. [107] described the role of miR-195, miR-24-2 and miR-365-2, in regulation of the anti-apoptotic protein Bcl-2. Their results revealed how the ectopic expression of these microRNAs led to a drop in Bcl-2 levels and conferred MCF7 cells a greater sensitivity to etoposide treatment.

Collectively, the findings of these studies suggest the importance of mitomiRs in regulating apoptosis and how their inhibition/overexpression could increase treatment sensitivity in some patients, especially those with TNBC, in whom therapeutic options are limited.

#### 3.3.3. Impact of the Mitochondria Components in the Carcinogenic Process

The mitochondrion is housed within a double membrane where a large number of arranged structures occur, including respiratory chain components, transporters like porins, and translocases and ribosomes so that they point towards the inside of the mitochondrion. The inner and outer mitochondrial membranes are separated by an intermembrane space where ions and other molecules needed for its functions are located [108]. In cancer, it has been described that some of these components may be altered, contributing to the process of carcinogenesis [109].

The inside of the mitochondrion, so-called mitochondrial matrix, is where water, ions, enzymes, metabolites and DNA and RNA molecules are found. The mitochondrial genome is a double strand of maternally inherited DNA of 16.6 kb that codes for 37 genes: 2 rRNAs, 22 tRNAs and 13 proteins of the mitochondrial oxidative phosphorylation complex, although nuclear DNA codes for a large proportion of proteins of the different complexes [110]. Both genomic and mitochondrial DNA (mtDNA) can be affected by several mutations in cancer, while the latter is more sensitive to mutations or oxidative stress-induced damage due to its greater susceptibility to free radicals, partially due to its lower repair capacity too [111], it has been established that, in effect many tumor cells show somatic mutations in mtDNA, as well as modifications in its contents. Further, there is continuous interplay between the expression of mitochondrial and nuclear genes, and this communication in the cell is dynamic [112]. The mitochondrion is thus able to control various cell processes including ATP production through cell respiration, the synthesis of cell metabolites, redox potential maintenance, regulation of ions such as calcium, and apoptosis. Further, the intracellular localization itself of the mitochondrion may determine itself which of these functions are fulfilled [113], reflecting the great complexity of this organelle.

#### 3.3.4. MicroRNAs in Mitochondria Biogenesis and Degradation

Levels of mitochondria are regulated by two opposing processes, mitochondrial biogenesis and mitophagy, a selective autophagy mechanism of dysfunctional mitochondria. In cancer, it is known that both these processes are modified due to the interactions of different factors such as metabolic reprogramming, heterogeneity, tissue type, microenvironment, and tumour stage [114].

Peroxisome proliferator-activated receptor gamma coactivator 1-α (PGC-1α) is a key regulator of mitochondrial biogenesis. A possible role of this factor in breast cancer has been proposed whereby fatty acid metabolism, oxidative stress levels and mitochondrial respiration are regulated via its binding to different transcription factors [115]. PGC-1α is also able to bind to and activate the oestrogen related receptor alpha (ERRα), which is found overexpressed in all the different molecular subtypes of breast cancer [116]. In some studies, direct correlation has been observed between PGC-1α levels and the formation of metastases [117,118]. In the cell, PGC-1α shows a dynamic localization and distribution pattern [119] and mitomiR could be responsible for this. Studies have shown that the inhibitors of PGC-1α expression, the two miR-485 isoforms, miR-485-3p and miR-485-5p, along with miR-217, are found diminished in breast cancer. Further, direct correlation was observed between this finding and a greater proliferation, invasive and metastatic capacity of these cells [120,121]. PGC-1α inhibition is thus a promising therapeutic approach to prevent metastasis.

The oncogene c-Myc is found modified in 30–50% of advanced breast tumours and is especially important in TNBC tumours and in tumours resistant to chemotherapy [122]. Among the diverse functions of c-Myc in the tumour cell, it is known that it modulates the expression of several nuclear genes coding for mitochondrial proteins and it also plays an important part in regulating mitochondrial biogenesis [123]. In addition, it promotes glutaminolysis in the mitochondrion, which consists of the incorporation of glutamine in the tricarboxylic acid cycle (TCA) via the actions of the enzyme glutaminase, as a key process in the reprogramming of tumour metabolism [124]. Interestingly, Zhao and Jiang [125] reported the downregulation of miR-4282 in invasive breast cancer cells and that its overexpression diminished c-Myc levels.

Mitophagy is another event that is found modified in cancer facilitating the survival and resistance of some tumour cells to different therapies [126]. Zhang et al. [28] showed that miR-1 is differentially downregulated in breast cancer stem cells (BCSCs) compared to non-stem cells. These authors also described that miR-1 overexpression in BCSC induces mitophagy through its binding to the 3′UTR region of the mRNA of mitochondrial inner membrane organizing system 1 (MINOS1) and of glycerol-3-phosphate dehydrogenase 2 (GP2), or via its direct binding to the protein leucine-rich pentatricopeptide-repeat containing (LRPPRC). These proteins play an important role in the organization and stability of the inner membrane, in the metabolic reprogramming that tumour cells undergo and in the maintenance of mitochondrial mRNA stability along with the translation of its products [127,128,129]. Low miR-1 levels could contribute to maintaining the tumour stem cell phenotype as a promising target for the treatment of breast cancer and prevention of recurrence.

#### 3.3.5. MicroRNAs in Mitochondria-Nucleus Interactions

MicroRNAs are crucial for the epigenetic mechanisms described in breast cancer, exerting their modulating actions at different levels. As described above, there is continuous communication between the nucleus, cytoplasm and mitochondrion. This crosstalk may be anterograde (nucleus-mitochondrion) or retrograde (mitochondrion-nucleus) and seems to be mediated by a series of proteostatic and transcriptional regulators [130] such as mitomiR.

Sripada et al. [131] examined the role played by miR-4485 in regulating mitochondrial functions in breast cancer. These authors noted that these microRNAs were translocated in smaller or greater measure to the mitochondrion in response to different cellular stress conditions. Hence, miR-4485 increased with the presence of TNF-α, and its import was diminished in other situations such as during endoplasmic reticulum stress, this being a good example of anterograde communication. In addition, miR-4485 was observed to regulate the processing of 16 S mitochondrial pre-rRNA, along with the translation of other downstream transcripts. Finally, they also detected diminished miR-4485 levels in the tissues of patients with breast cancer and observed that this microRNA modulated mitochondrial complex I activity, ATP production, ROS levels, caspase 3 and 7 activation and apoptosis. Interestingly, they concluded that the transfection of breast tumour cells with miR-4485 led to reduced glycolytic enzyme expression and cell malignization as a promising target for breast cancer treatment.

In parallel, Carden et al. [132] examined the role of miR-663 by determining in vitro levels of this microRNA in Rho^0^ breast cancer cell lines with and without mtDNA. Results indicated reduced levels in cell lines lacking mtDNA, promoting cell proliferation and tumorigenesis. In an elegant experiment, these authors were able to rescue normal miR-663 levels by adding mtDNA, thus demonstrating the role of this microRNA in retrograde signaling. These authors also detected direct correlation between levels of this microRNA and patient survival. In addition, they confirmed the importance of miR-663 regulation in the expression of the different respiratory chain components and how these levels could be regulated epigenetically by reactive oxygen species (ROS).

Mitochondrial DNA variations play an important role in cancer. In effect, it is known that many tumour cells show somatic mtDNA mutations along with content modifications. For example, Guerra et al. [133] reported a reduction in the quantity of mtDNA in initiating tumour cells, which seem to play a key role in cancer recurrence subsequent to chemotherapy. However, it remains to be clarified whether these mutations drive this change or are simply its consequence [134]. Singh et al. [135] addressed how germinal mutations in polymerase gamma (POLG1) DNA induced mutations or loss of mtDNA, along with a drop in oxidative phosphorylation conferring an increase in glucose consumption, decrease in ATP production and greater invasiveness. Notably, their results showed that these characteristics were epigenetically controlled by a set of mitomiR, whose normal expression levels were restored in the absence of these mutations.

#### 3.3.6. MitomiR in Mitochondrial Fission and Fusion Dynamics

Mitochondrial dynamics also seems of great importance in the biology of tumour cells and has been linked to a wide array of cell processes such as cell proliferation, survival, and metastasis [136]. Mitochondria are very dynamic organelles that undergo constant processes of fusion and fission [137]. Through fission, an original mitochondrion gives rise to two daughter mitochondria. The process is regulated by a series of proteins, among which dynamin-related protein 1 (Drp1) plays an especially important role. Conversely, fusion is the process whereby mitochondrial outer membranes fuse together and features the role of mitofusins 1 and 2 (MFN-1 and 2) [138]. The contribution of different mitomiR to the control of mitochondrial dynamics has also been described. For example, it has been reported that in breast cancer, the downregulation of miR-195 intervenes in mitochondrial dynamics, as another of its targets is MTN2, which escapes its inhibition [139]. Wang et al. [140] also described the role of oncomiR-484 in regulating levels of Fis1, a protein located at the outer mitochondrial membrane with a possible role in mitochondrial fusion and whose levels increase when the cell enters into apoptosis [141,142]. Interestingly, Zearo et al. [143] identified significant differences in levels of miR-484 in the serum of healthy patients compared to those with early breast cancer, suggesting the possible involvement of this microRNA in the mitochondrial changes that take place in tumour cells in early disease stages.

#### 3.3.7. Mitochondria and Immune System Interactions

Immune system modulation by the tumour cell is key to its development and progression such that an inflammatory microenvironment and evasion of the immune system are important for the tumour. Some authors argue that while genetic damage may be “the spark that starts the fire”, inflammation is the fuel that keeps it going [144]. The mitochondrion is thought to contribute to inflammation due to its essential role in signaling cell damage through damage-associated molecular patterns (DAMPs). These DAMPs can act as inducers, sensors, and mediators of stress both inside and outside the cell as they are responsible in part for the immune system response that occurs in cancer [145]. Among these DAMPs derived from the mitochondrion are mtDNA, ATP or cytochome C. One of mechanisms through which DAMPs can be expelled to the tumour’s microenvironment is via necrosis or necroptosis. Recent studies have shown how both processes are crucial for the regulation of angiogenesis, metastasis and immune system interactions [146,147]. Kapetanovic et al. [148] associated elevated levels of DAMPs and the onset of low-grade chronic inflammation with elevated levels of interleukin 6 (IL-6) and C reactive protein (CRP). Both these markers have been associated with the development of numerous age-related diseases, including cancer.

MiR-200a is another microRNA found overexpressed in breast cancer [149]. One of the targets of this microRNA is mitochondrial transcription factor A (TFAM) whose significance has been described in various malignancies including breast cancer [150,151,152]. TFAM is a primary transcription factor in the mitochondrion, with described key roles in the stability, replication, and transcription of mitochondrial DNA [153]. Recently, Yang et al. [154] identified TFAM as a DAMP released from the mitochondrion in apoptotic cells that is essential for the cell response mediated by the immune system. Hence tumour cells could benefit from TFAM blockade as a way to evade the immune system. Further, Fan et al. [155] reported that miR-199a-3p was also able to inhibit TFAM in breast cancer cells, promoting their resistance to cisplatin.

## 4. From Basic to Translational Research. MicroRNAs and the Management of Patients with Breast Cancer: Present and Future

Because of their important role as key regulators of multiple cell targets, microRNAs potential applications have arisen not only for basic but also for translational research. Currently, three major areas are being developed for cancer’s clinical management: Diagnosis, prognosis markers and therapeutic approaches [156].

The identification of expression profiles of the different microRNAs is one of the most promising advances in precision medicine for patients with cancer [157,158].

MicroRNAs can be detected and measured in blood using methods that are non-invasive for the patient. Notably, it is known that the study of a patient’s serum microRNA expression profile is more reliable and sensitive than the study of a single microRNA type [159]. Further, as described previously, some microRNAs can be examined by analysing extracellular vesicles such as exosomes, measurable in body fluids such as serum, and these methods are especially useful for the detection and use of these microRNAs [160].

The determination of microRNAs as prognostic and diagnostic markers of tumour progression gains growing support [161]. In a systematic review by Adhami et al. [162], it was shown that breast cancer cells feature increased levels of miR-21 and miR-210 along with a lower expression of miR-145, miR-139-5p, miR-195, miR-99a, miR-497 and miR-205, suggesting their prognostic value and utility for an early diagnosis in the patient. Recently He et al. [163] examined the validity of some of these microRNAs to assess survival in patients with breast cancer. Their results pointed to correlation between levels of miR-21 and miR-22 and patient survival and to a complex network comprising 5 microRNA and a series of essential factors with the same ability to predict survival. Braga et al. [164] showed how the detection of miR-125b and the methylation of at least one of its target genes was sufficient to diagnose breast cancer in a tissue biopsy.

The study of the different expression profiles of microRNAs according to the tumour’s molecular profile is an interesting topic with implications for the management of breast cancer. Some of the most important microRNAs with suppressor functions have been recently identified by Dastmalchi et al. [165]. Interestingly, Haakensen et al. [166] did not find significative differences in the transition from in situ tumours to invasive tumours, thus denoting a greater impact of the use of microRNAs when the molecular classification is used rather than their invasiveness stratification. miR-142-5p, miR-320a, and miR-4433b-5p were found differentially expressed in extracellular vesicles in patients with breast cancer compared with control subjects. In an interesting study, the expression of miR-142-5p and miR-320a was detected in patients with luminal A breast cancer but not in those with TNBC or controls. In advanced tumours, diminished miR-142-5p and R-150-5p were detected (stage III) while the decreased expression of miR-142-5p and miR-320a was related to a larger tumour size [167]. Wu et al. [168] reported that the expression of the three microRNAs miR-21, miR-659-5p and miR-200b-5p was a prognostic biomarker in patients with TNBC. Stevic et al. [169] also found in exosomes obtained from TNBC and HER2+ patients before and after undergoing chemotherapy, that miR-155 and miR-301 were the best microRNAs at predicting a pathological complete response (pCR). The study of lncRNA and its interaction with microRNA is another growing area of research on breast cancer treatment. Tripathi et al. [170] described that the identification of the network lncRNA → microRNA → mRNA could be key to analyse the regulation of microRNA in breast cancer and the products that regulate it, representing an interesting therapeutic target for future studies. In this line, the effectiveness of studying the expression profiling of mitomiRs has been shown in the diagnosis of heart failure, where mitochondrion impairment plays a prominent role of the pathophysiology [171]. In the case of breast cancer, the best way mitomiRs may be studied is through a biopsy. On the other hand, it has been demonstrated that exosomes are able to carry not only microRNAs, but also AGO-2, Dicer and even mtDNA [172] suggesting the possible role that exosomes may have as a factory and transport of mitomiR amongst cells, emerging as a potential diagnosis marker. Further studies will be needed to validate the role of mitomiRs as diagnosis markers

MicroRNAs are also a therapeutic target in breast cancer and promising results have been obtained in numerous preclinical trials [173]. Gilam et al. [174] showed that the overexpression of some microRNAs like miR-96 or miR-182 may diminish the migration and invasion capacity of breast cancer tumour cells by reducing levels of the protein Palladin. Further, Yi et al. [175] recently developed a new high-performance platform for the production of bioengineered microRNA agents (BERA). The platform was successful at producing a humanized molecule directed at miR-328 (hBERA/miR-328) and is therefore an important resource for future therapies involving microRNA inhibition. Developments in some areas such as in nanomedicine are allowing for greater specificity and efficacy of breast cancer treatments. Several studies have shown that microRNAs or small interfering RNAs (siR) used in these systems are a promising target in breast cancer [176,177]. In this regard, Kardani et al. [178] designed a nanoparticle comprised of gold particles and an miR-155 antagonist to inhibit its action. Their results revealed a significant reduction in the levels of this microRNA and higher levels of TP53INP1 mRNA, inhibiting the proliferation and promoting the apoptosis of these tumour cells. Notwithstanding it is also true that, therapeutic market of microRNAs is less established in breast cancer management than the other areas, mainly focused on microRNA mimics and antagomir products [156] In fact, these strategies are only designed to treat hepatitis C and, to our knowledge there is still no pharmaceutical products targeting breast cancer cells. Although there is still a long road to cover, mitomiRs could be a promising therapeutic target of future research oriented to inhibit oncomicroRNAs or act as tumor suppressors microRNAs, directly regulating mitochondria function, which may be critical to modulate or to stop a broad range of carcinogenesis processes.

## 5. Conclusions

**Figure 3:**
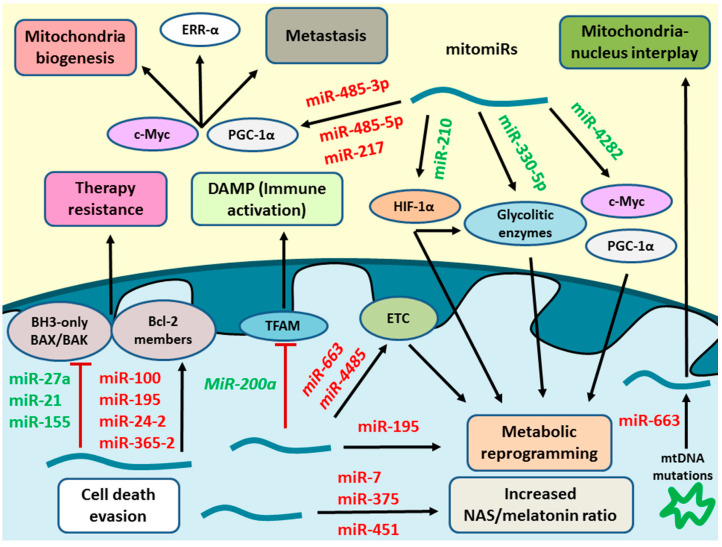
A general overview of some of the most important mitomiRs in breast cancer. As represented in the figure, mitomiRs may play a prominent role in multiple carcinogenic processes by their upregulation (Green) and downregulation (Red). Several mitomiRs are implicated in metabolic reprogramming in breast cancer cells at various levels such as Warburg effect, electron transport chain (ETC) lipid or amino acid metabolism. MitomiRs can achieve these goals through their interaction with cytosolic or mitochondrial targets. Some of the products regulated by mitomiRs are critical controlling a broad range of tumorigenic mechanisms like c-Myc or PGC-1α. Moreover, it is known that mitomiRs may inhibit proapoptotic proteins or the expression of vital mitochondrial regulators like TFAM, thus promoting therapy resistance to chemotherapeutic agents and modulating the interaction of the tumor with immune system. Other microRNAs have been shown to take part in the nucleus-mitochondria interactions, as is the case of miR-663, directly influenced by mitochondrial DNA mutations or miR-4485, whose levels are modulated by the nucleus and TNFα, acting at various stages in mitochondria. Finally, the potential of mitomiRs as therapeutic targets must be considered in future clinical management of breast cancer, as it is the case of miR-7, miR-375 and miR-451 in the metabolism of melatonin, a toxicological product for breast cancer cells. Further microRNAs agonists or antagonists should be developed to properly target mitomiRs.

MitomiRs are a subgroup of microRNA defined by targeting mitochondrial or cytosolic products in charge of modulating mitochondrion functionality. As described in this article, these molecules have a great impact on the carcinogenesis process in breast cancer due to their direct or indirect actions on mitochondria and in conferring resistance to conventional treatments (Figure 3). Although it is still at the beginning, mitomiRs show a huge potential as therapeutic targets and as prognostic and diagnostic markers (Table 1). However, most of these studies have been tested only in vitro and in vivo models, and further research is needed to approach the translation for humans.

There is a need to gain deeper insight into why these mitomiR undergo modification in cells and to understand the mechanisms whereby the mitochondrion is able to import and regulate mitochondrial levels of these microRNAs. Finally, other lines of investigation need to examine the expression profiles of these mitomiR in each molecular subtype of breast cancer and in male patients. This type of study is needed to improve our understanding of the molecular biology of these tumors in the search for ever more effective and precise therapies and systems directed at these targets.

Despite this, we still have a long way to go, these studies reveal the importance of an adequate understanding of the role of microRNAs as a way of advancing in the treatment of this common cancer.

## Figures and Tables

**Table 1:** Main mitomiRs described in breast cancer.

MicroRNA	Expression	Target	Implications	Potential Use	Reference
miR-1	Downregulated	Mitochondrial (MINOS-1, LRPPRC) Cytosol (GP-2)	Mitophagy evasion by BCSCs	Therapeutic target: Inducing mitophagy of stem cells In vitro and In vivo	Zhang et al., 2019 [28]
miR-7 miR-375 miR-451	Downregulated	Cytosolic 14-3-3ζ, protein	Increased NAS/melatonin ratio: Modified melatonin metabolism and cell survival	Therapeutic and prevention target in vitro	Anderson 2019 [86]
Let-7	Downregulated	Mitochondrial PDK-1 Apoptotic proteins	Metabolic reprogramming Increased sensitivity to doxorubicin in TNBC	Therapeutic target Causing mitochondrial ROS production. In combination with doxorubicin, increases sensitivity to the drug. Biomarker of invasiveness	Serguienko et al., 2018 [70] Marques et al., 2018 [87]
miR-21	Upregulated	Mitochondrial apoptotic proteins	Apoptosis evasion	Therapeutic target: Downregulation by metformin may be correlated with the suppression of cell proliferation and migration Early diagnosis Survival prediction TNBC specific prognosis biomarker	Sharma & Kumar 2018 [100] Adhami et al., 2018 [162] He et al., 2019 [163] Wu et al., 2020 [168]
miR-24-2 miR-365-2	Downregulated	Mitochondrial Bcl-2 protein	Apoptosis evasion Etoposide resistance	Therapeutic target: Negative regulator of BCL2, reducing levels of BCL2 protein and increasing apoptosis	Singh & Saini 2012 [107]
miR-27a	Upregulated	Mitochondrial proapoptotic protein (BAK)	Apoptosis evasion Increased proliferation and metastasis	Therapeutic target: Knockdown increases sensitivity of T-47D cells to cisplatin	Zhou et al., 2016 [99]
miR-100	Downregulated	Mitochondrial antiapoptotic protein HAX-1	Apoptosis evasion Cisplatin resistance	Therapeutic target: HAX-1 increases chemosensitivity of breast cancer	Wu et al., 2018 [102]
miR-125b	Downregulated	Mitochondrial apoptotic proteins	Apoptosis evasion Doxorubicin resistance	Coadjuvant therapy: miR-125b in combination with doxorubicin leads to loss of mitochondrial membrane potential Diagnosis	Xie et al., 2015 [104] Hu et al., 2018 [105] Braga et al., 2020 [164]
miR-133a	Downregulated	Mitochondrial UCP-2	Metabolic reprogramming Doxorubicin resistance	Therapeutic target: miR133a/UCP-2 signaling for chemotherapy resistant	Yuan et al., 2015 [73]
miR-155	Upregulated	Mitochondrial apoptotic proteins	Apoptosis evasion	Therapeutic target: miR-155 may be downregulated by metformin, which allows suppression of cell proliferation and migration Pathologic complete response Nanomedicine target: Gold Nanoparticle-Aptamer-anti-miR-155 development	Sharma & Kumar 2018 [100] Stevic et al., 2018 [169] Kardani et al., 2020 [178]
miR-181c	Upregulated	Mitochondrial MTC01 Cytosolic AMPK	Metabolic reprogramming Cell signalling pathways	Therapeutic target: Potential target to inhibit both NFE2L2/NRF2 and AMPKα in breast cancer cells	Jung et al., 2017 [75]
miR-195	Downregulated	Mitochondrial CYP27B1, Bcl-2 MTN-2 Cytosolic lipogenesis enzymes	Metabolic reprogramming Apoptosis evasion Modified mitochondrial dynamics Etoposide resistance	Therapeutic target: miR-195 targets BCL inducing apoptosis. It also reduces proliferation, invasion, and migration. Prognosis Early diagnosis	Singh et al., 2015 [88] Singh & Saini 2012 [107] Purohit et al., 2019 [139] Adhami et al., 2018 [162]
miR-199a-3p miR-200a	Upregulated	Mitochondrial TFAM	Mitochondrial genome instability Immune interactions Cisplatin resistance	Therapeutic target: BC cells growth and mitochondrial DNA copy number regulations	Yao et al., 2014 [149] Yao et al., 2018 [152] Fan et al., 2017 [155]
miR-210	Upregulated (by HIF-1α)	Mitochondrial and cytosolic targets	Metabolic reprogramming DNA damage Apoptosis evasion Angiogenesis Cell cycle alterations	Therapeutic target: overexpression makes cells more susceptibleto killing by 3-bromo-pyruvate Prognosis Early diagnosis	Ivan & Huang 2014 [68] Adhami et al., 2018 [162]
miR-217 miR-485-3p miR-485-5p	Downregulated	PGC-1α	Mitochondrial biogenesis Increased proliferation Invasion and metastasis	Therapeutic target: In vitro, overexpression of miR-485-3p and miR-485-5p suppressed mitochondrial respiration and potential for cell migration and invasión. In vivo, they inhibited spontaneous metastasis of BC cells.	Lou et al., 2016 [120] Zhang et al., 2017 [121]
miR-223	Downregulated	Mitochondrial HAX-1	Apoptosis evasion by TRAIL in TNBC	Therapeutic target: Overexpression increases sensitivity of TNBCSCs to TRAIL-induced apoptosis	Sun et al., 2016 [103]
miR-330-5p	Upregulated (by SNHG3)	Mitochondrial and cytosolic metabolic enzymes: PKM2	Metabolic reprogramming	Therapeutic target: When increased, SNHG3 knockdown in CAF-secreted exosomes suppressed glycolysis metabolism and cell proliferation	Li et al., 2020 [71]
miR-484	Upregulated	Mitochondrial Fis-1	Mitochondrial dynamics Apoptosis evasion Cancer development	Therapeutic target: In vivo models with high or low levels of miR-484 correlates with reduced or enhanced mitochondrial fission, apoptosis, and myocardial infarction	Wang et al., 2012 [140]
miR-519d	Downregulated	Mitochondrial antiapoptotic protein MCL-1	BCSC sensitivity to cisplatin reduced	Coadjuvant therapy: Induced expression of miR-519d in T-47D-cancer stem cells increased their sensitivity to cisplatin via apoptosis	Xie et al., 2017 [106]
miR-663	Downregulated (by ROS)	Mitochondrial ETC components	Mitochondrion-nucleus signalling; increased proliferation, tumorigenesis	Therapeutic target: antimir-663 increased in vitro cellular proliferation and promoted tumor development in vivo	Carden et al., 2017 [132]
miR-3677	Upregulated	TLE3	Metabolic reprogramming	Therapeutic target: Silencing TLE3 may suppress proliferation and migration in BC cells.	Peng et al., 2020 [78]
miR-4282	Downregulated	Nuclear c-Myc	c-Myc driven pathways; glutaminolysis chemotherapy resistance	Therapeutic target: In silico and in vitro analyses showed that Myc might be the target of miR-4282 in BC	Zhao et al., 2018 [125]
miR-4485	Downregulated	Mitochondrial pre-rRNA 16 S ETC complex I Others	Abnormal ATP production and mitochondrial RNA processing; ROS regulation; apoptosis evasion	Therapeutic target: In vitro miR-4485 downregulated glycolytic pathway genes and decreased proliferation of BC cels. In vivo model, expression of this miR decreased the tumorigenicity	Sripada et al., 2017 [131]

This table shows expression levels of microRNAs, their most important mitochondrial and cytosolic targets and the effects of their dysregulation in the tumour cell according to the literature reviewed. MicroRNAs can directly modulate mitochondrial function through the regulation of mitochondrial products or by controlling other key elements that control this organelle such as c-Myc, PGC-1α or 14-3-3ζ proteins. In addition, the table summarises potential uses of the main mitomiRs established in the different studies.

## Abbreviations

ER	Oestrogen Receptor
PR	Progesterone receptor
miR	MicroRNA
mitomiR	Mitochondrial microRNA
pri-microRNA	Primary microRNA
DGCR8	DiGeorge Syndrome Critical Region 8
pre-microRNA	Precursor microRNA
XPO5	Exportin 5
ds-microRNA	Double-stranded microRNA
RISC	RNA-induced silencing complex
TRBP	Trans-activation response RNA-binding protein
AGO	Argonaute
MRE	MicroRNA response element
mRNA	Messenger RNA
IPO8	Importin 8
mtDNA	Mitochondrial DNA
PET	Positron emission tomography
HIF-1α	Hypoxia-induced factor 1α
PDK1	Pyruvate dehydrogenase kinase 1
CAF	Cancer-associated fibroblasts
PKM2	Pyruvate Kinase M2
LncRNA	Long non-coding RNA
NFE2L2	Nuclear factor (erythroid-derived 2)-like 2
MTC01	Subunit 1 of cytochrome C oxidase gene
TLE3	Transducin-like enhancer of split3
GPCR	G protein coupled receptor
NAS	N-acetyl serotonin
CYP27B1	Cytochrome P450 family 27, subfamily B, polypeptide 1
ACACA	Acetyl-CoA carboxylase 1/alpha
FASN	Fatty acid synthase
HMGCR	3-Hydroxy-3-methylglutaryl-CoA reductase
mPTP	Mitochondrial permeability transition pore
MOMP	Mitochondrial outer membrane permeabilization
APAF-1	Apoptotic protease activation factor 1
TRAIL	Tumour necrosis factor-related apoptosis-inducing ligand
ERRα	Oestrogen related receptor alpha
TCA	Tricarboxylic acid cycle
BCSCs	Breast cancer stem cells
ROS	Reactive oxygen species
POLG1	DNA polymerase gamma
Drp1	Dynamin-related protein 1
MFN-1/2	Mitofusins 1 and 2
DAMPs	Damage-associated molecular patterns
IL-6	Interleukin 6
CRP	C reactive protein
TFAM	Mitochondrial transcription factor A
(pCR)	Pathological complete response
